# A high resolution spatial population database of Somalia for disease risk mapping

**DOI:** 10.1186/1476-072X-9-45

**Published:** 2010-09-14

**Authors:** Catherine Linard, Victor A Alegana, Abdisalan M Noor, Robert W Snow, Andrew J Tatem

**Affiliations:** 1 Spatial Ecology and Epidemiology Group, Department of Zoology, University of Oxford, Tinbergen Building, South Parks Road, Oxford, OX1 3PS, UK; 2 Biological Control and Spatial Ecology, Université Libre de Bruxelles CP 160/12, av. F.D. Roosevelt 50, B-1050 Brussels, Belgium; 3 Malaria Public Health and Epidemiology Group, Centre for Geographic Medicine, KEMRI - University of Oxford - Wellcome Trust Collaborative Programme, Kenyatta National Hospital Grounds (behind NASCOP), P.O. Box 43640-00100, Nairobi, Kenya; 4 Centre for Tropical Medicine, Nuffield Department of Clinical Medicine, University of Oxford, CCVTM, Oxford OX3 7LJ, UK; 5 Department of Geography, 3141 Turlington Hall, University of Florida, Gainesville, Florida, 32611-7315, USA; 6 Emerging Pathogens Institute, University of Florida, Gainesville, Florida, 32610-0009, USA; 7 Fogarty International Center, National Institutes of Health, Bethesda, MD 20892, USA

## Abstract

**Background:**

Millions of Somali have been deprived of basic health services due to the unstable political situation of their country. Attempts are being made to reconstruct the health sector, in particular to estimate the extent of infectious disease burden. However, any approach that requires the use of modelled disease rates requires reasonable information on population distribution. In a low-income country such as Somalia, population data are lacking, are of poor quality, or become outdated rapidly. Modelling methods are therefore needed for the production of contemporary and spatially detailed population data.

**Results:**

Here land cover information derived from satellite imagery and existing settlement point datasets were used for the spatial reallocation of populations within census units. We used simple and semi-automated methods that can be implemented with free image processing software to produce an easily updatable gridded population dataset at 100 × 100 meters spatial resolution. The 2010 population dataset was matched to administrative population totals projected by the UN. Comparison tests between the new dataset and existing population datasets revealed important differences in population size distributions, and in population at risk of malaria estimates. These differences are particularly important in more densely populated areas and strongly depend on the settlement data used in the modelling approach.

**Conclusions:**

The results show that it is possible to produce detailed, contemporary and easily updatable settlement and population distribution datasets of Somalia using existing data. The 2010 population dataset produced is freely available as a product of the AfriPop Project and can be downloaded from: http://www.afripop.org.

## Background

Since the fall of the central government in 1989, Somalia has been suffering from 18 years of conflict and civil war that has resulted in a series of humanitarian disasters. Thousands of Somali have been externally and internally displaced and millions have been deprived of basic health and social services [1]. Attempts are being made to reconstruct the health sector but with the exception of the relatively peaceful northern region, the current political situation in Somalia poses a major obstacle for the establishment of a comprehensive health care system. The situation has inevitably resulted in an almost complete lack of any systematic, comprehensive nationwide systems of vital or health information.

Defining the extent of infectious diseases as a public health burden and their distribution in time and space are critical to scoping the financial requirements, setting a control agenda and monitoring [2]. However, any approach that requires the use of modelled disease rates requires reasonable information on the resident population for the years one is intending to estimate risk. Where risks of disease are over-distributed in space, such as is the case for most vector borne diseases, population distributions and counts must be resolved to higher levels of spatial detail than large regional estimates. Detailed and spatially disaggregated population data are essential resources in the assessment of the number of impacted people for planning health service delivery and for decision-making processes related to developmental or health issues [3-6].

Whilst high-income countries generally have extensive mapping resources and demographic data at their disposal, in low-income countries relevant data are either lacking or are of poor quality. In Somalia, the last national census was undertaken in 1987 [7]. The major changes in population size and distribution that are occurring in Somalia undermine the fidelity of the population parameters from the 1987 census to derive contemporary estimates. In addition, census population counts are reported in large spatial units (regions or districts), when the known spatial pattern of disease risk is generally highly focal and spatially heterogeneous. The coarse spatial resolution of Somalia census data therefore limits its potential use for resource allocation and disease burden estimation.

Modelling techniques for the spatial reallocation of populations within census units have been developed based on ancillary datasets, such as transport networks, urban extents and slopes [3,5,8,9], but work has also shown that, unless these datasets are complete and of finer detail than the census data, such modelling techniques can be detrimental to mapping accuracies [5]. Analyses have also shown that land cover (LC) information derived from satellite imagery can be valuable in redistributing aggregated census counts to improve the accuracy of national-scale gridded population data in East Africa [10-13]. The intrinsic link between human population distribution and LC, particularly settlements, means that such data likely offer the best opportunity for improved population distribution modelling. Here we extend the methods developed by Tatem et al. [12], and recently refined [13], to model population distributions and densities at a fine spatial resolution in order to provide an evidence base for refining infectious disease mapping and for planning health service delivery in Somalia.

## Data

### Land cover and other GIS data

The Africover programme, operational in the 1995-2002 period, provided a multipurpose and consistent LC GIS database of 10 countries in East Africa. The Africover data uses land cover classification software (LCCS) for creating standardized and harmonized LC classes. This allows easy aggregation of LC classes based on a hierarchy of LC class detail, and allows comparison of LC classes across countries and regions. The full resolution Africover dataset for Somalia was acquired [14] and the 99 individual classes were aggregated to a more generic 22 classes to provide a consistent legend across the entire region. Secondly, a dataset depicting urban and settlement polygon outlines from detailed imagery was acquired from the GeoTerraImage Consultancy [15]. This dataset was principally derived from 2005 Landsat imagery, through a combination of conventional on-screen interpretation and hierarchical clustering techniques, often involving the use of area-specific geographic masks. Industrial area delimitations for the main cities were also provided. In addition, roads and rivers data were obtained from Vector Map level Zero (VMAP0) dataset to aid testing and accuracy assessment.

### Settlement locations

In 2008, settlement point location data from different NGOs and UN agencies working in Somalia, including the United Nations Development Programme (UNDP), the German Agency for Technical Cooperation (GTZ), the Kenya Medical Research Institute (KEMRI), the Food Security Analysis Unit (FSAU), and the UN Office for the Coordination of Humanitarian Affairs (OCHA), were assembled into a single file. The dataset consists of 11,413 settlement locations divided in 8 categories: national capital (1); regional capital (17); district capital (57); town (13); part of town (186); settlement (9852); IDP camp (535); and temporary nomadic settlement (752). These 8 categories were aggregated into three classes: IDP camps, rural settlements and towns (this last group including national, regional and district capitals, towns and parts of towns). The nomadic settlements were removed from the database because of their temporary locations, but nomads were included in the population estimates. Some of the settlement locations include population size estimations, from different sources: KEMRI, GTZ and UNDP. When combining these differing sources of estimates, half of the settlement locations (49.4%) had a population size attribute (38% of towns, 52% of rural settlements and 0.9% of IDP camps).

### Population data

Population count data were available at administrative level 2 (district) in Somalia. There are 74 districts, giving an average spatial resolution (ASR) of 93 km. The ASR measures the effective resolution of administrative units in kilometers, and is calculated as the square root of the land area divided by the number of administrative units [16]. The OCHA provided population estimates by district for the year 2005. These are the most recent population data available for Somalia, as the last nationwide census dates back to 1987.

### Population data for the Afgooye corridor

The UN High Commission for Refugees (UNHCR) and OCHA regularly provide updated estimates of refugee camp sizes and population movements in Somalia. The Afgooye corridor is a particularly dynamic area near Mogadishu, where more than 100 IDP camps are located, with a total of 366,000 refugees as of January 2010 [17]. UNHCR and OCHA also provide recent estimations of surface area covered by IDP camps and population counts in 5 sub-areas of the Afgooye corridor [17,18]. These data were obtained for use within the modelling process.

### Detailed training data

Detailed census data at administrative unit level 6 (enumeration area) from the 1999 population and housing census in Kenya, with corresponding administrative unit boundaries for 59 of the 69 Kenyan districts, were also obtained [19] to provide guidance on typical population densities by land cover type in the East Africa region.

## Methods

Most population modelling methods essentially involve some form of re-distribution of aggregate census counts using ancillary datasets at finer spatial detail that are known to influence human population distribution. Here, the district level population count data were redistributed at a finer spatial scale using all the available information contained in the datasets described above. Specifically, the Africover LC dataset was first adapted to accommodate the more precise and detailed mapping and locational information on settlements provided by the Landsat-derived settlement polygons, the settlement points and refugee camps, all described above. Next, LC specific weights were derived based on information on population sizes from the settlement points and from the detailed Kenyan census data where the same LC classes exist (as described in [12]). These calculated densities were then utilised as weightings to redistribute population by settlement and LC type that were unaccounted for by existing settlement population size data. More detail on the full process is outlined below.

### Land cover data refinement

The Africover urban class, which typically overestimates settlement extent size [11,12], was first removed and the surrounding classes expanded equally to fill the remaining space. The settlement location data and the Landsat-derived settlement polygons were then used to refine the 'urban area', 'rural settlement', 'refugee camp' and 'industrial area' classes. Given the clustered nature of populations across Somalia, ensuring that all known settlements were identified and mapped using information from all available datasets represented an important step.

1. Urban areas

The urban class refinement was mainly based on settlement locations classified as 'towns'. Town extents were mapped in three different ways according to available data: (i) the Landsat-derived settlement polygons were mapped when town location points could be mapped unambiguously onto a polygon, (ii) information on population size was used to provide an estimate of town extent, where just a single georeferenced location existed, and (iii) an average town extent of 1.04 km² - which corresponds to the average size of Landsat-derived settlement polygons intersecting with towns - was used for towns where just a single point existed and the population size was unknown. An urban extent map was derived from these town extents.

2. Rural settlements

A similar method as above was used to produce a rural settlement layer, based on rural settlement points: (i) the Landsat-derived settlement polygons were used when settlements could unambiguously be mapped onto a polygon, (ii) information on population sizes were used to provide an estimate of settlement extent where just a single georeferenced location existed, and (iii) a settlement extent of 10,000 m² (i.e. one pixel) was used for settlements where just a single point existed and the population size was unknown. We did not use the average size of rural settlements because only 2.7% of rural settlement points intersected Landsat-derived settlement polygons (in contrast to 69% for towns), which suggests that only the biggest settlements were detected in Landsat-derived settlement polygons database and that, using these, the average size would then likely be overestimated.

3. IDP camps

The 'refugee camp' class mapping was mainly based on settlement locations classified as 'IDP camp': (i) information on population sizes were used to provide an estimate of IDP camp spatial extent, and (ii) an average IDP camp extent of 0.04 km² - which was calculated based on the UN data for the Afgooye corridor - was used for IDP camps where the population size was unknown. IDP camp extents were assembled to form a 'refugee camp' map.

4. Industrial areas

The Landsat-derived industrial area delimitations were used to define an industrial area map.

The urban, rural settlements, refugee camps and industrial area maps were all overlaid onto the Africover dataset and the land covers beneath were replaced to produce a refined LC dataset.

### Land cover specific population densities

Relative per LC class population densities were defined for each class of the refined LC dataset. The average population density in urban areas and rural settlements were calculated based on the Landsat-derived settlement polygons combined with settlement population counts. Average population densities of 18,302 people/km² and 2,990 people/km² were calculated for urban areas and rural settlements, respectively. Typical population density in refugee camps was calculated based on available data for the Afgooye corridor. The Afgooye corridor is divided into 5 sub-areas, for which the UN OCHA estimated the surface area covered by IDP camps [18] and the UNHCR estimated population sizes [17]. From these data, we calculated an average population density of 77,199 people/km² in IDP camps. Zeros were attributed to classes with no human habitation such as water bodies, industrial areas and sand beaches.

The average population densities of the remaining LC classes were derived from the Kenyan census data, where significantly more accurate and detailed data on population distribution were available. The Kenyan Enumeration Area (EA) census data [19], which contain 46,034 EAs and has an ASR of just 3.21 km, provided a valuable dataset for calculating more accurate relative per LC class population densities than could be obtained from existing Somalia data. Moreover, all the Africover LC types found in Somalia are also present in Kenya. The average population density of one specific LC class was calculated based on EAs that record this LC class for the majority of their pixels, as outlined in [12] and [13]. As shown in [13], the extrapolation of LC specific population densities to neighbouring regions had a limited impact on population distribution model accuracies in Kenya. However, even if the relative values between population densities derived from Kenya are important, the absolute population density values can vary notably from one country to the other. Population densities derived from Kenya are expected to be overestimated because small settlements were not distinguished from major Africover classes in Kenya. Moreover, populations are much more clustered across the whole of Somalia due to the arid environment. We therefore varied the population densities derived from Kenya by scaling them by a sequence of weightings between 0 and 1 (with an increment of 0.01), while keeping the weights derived from Somalia data fixed. We tested the accuracy of population data produced based on each population density table by comparing predicted population with the observed population in towns and settlements from the location dataset with known populations. This provided a test of the repartition of populations between settlements/towns and other LC classes. The root mean square error (RMSE) was extracted for each population dataset. The LC specific population density table that produced the lowest RMSE was selected for the final population distribution model.

### Population distribution modelling

The per-LC class densities defined above were used as weightings to reallocate populations within Somali districts. Per-pixel population densities were adjusted to match the total population estimated by the UNDP (2005) in the administrative units that they belonged to. An estimate of population in 2010 was produced based on UN rural and urban growth rates for the 2005-2010 period, using the following equation: *P*_2010 _= *P*_2005_*e^rt^*, where *P_2010 _*is the required 2010 population within a pixel, *P_2005 _*is the population within the same pixel at year 2005, *t *is the number of years between year 2005 and 2010, and *r *is the average growth rate for rural pixels (2.21%) and urban pixels (4.17%) - these growth rates were taken from the UN World Urbanization Prospects Database, 2007 version [20].

### Comparison with existing datasets

**Figure 1:**
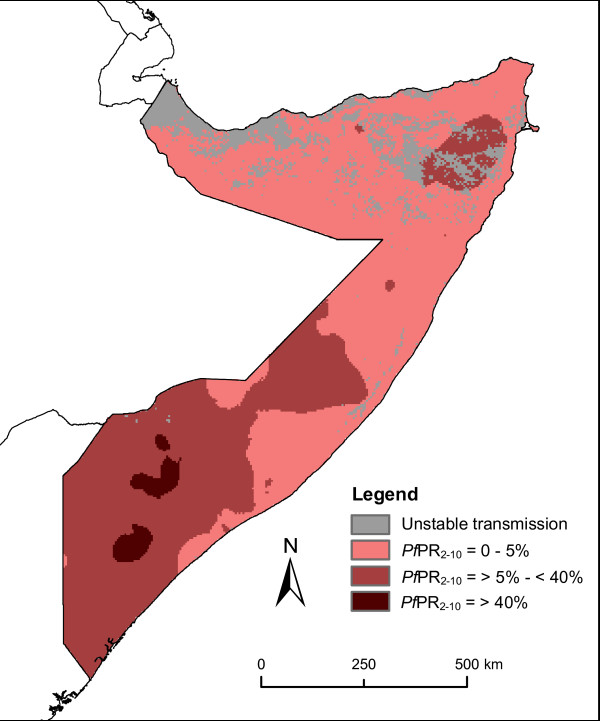
**Spatial distribution of *P. falciparum *malaria predictions stratified by endemicity class adapted from Hay et al. (2009) **[2].

Accuracy assessment of largescale population datasets is always challenging due to the use of all geographically-specific datasets to produce the population dataset, leaving little independent data for testing. However, simple comparison tests with existing gridded population datasets were undertaken. The 2008 version of LandScan [21] and the 2000 beta version of the Global Rural Urban Mapping Project (GRUMP) [22] are the most widely used population datasets, and were acquired and compared to the newly created dataset (AfriPop). Given the differing spatial resolutions, the tests should not be considered as formal accuracy assessments, but merely informative comparisons. To make the comparisons possible, population datasets were adjusted to the same year using UN growth rates [20] and resampled to 100 meters spatial resolution. Different methods were used to compare the AfriPop, GRUMP and LandScan datasets. Firstly, predicted population totals per district were compared to the UNDP population estimates for the year 2005. The three population datasets were adjusted to 2005 for this calculation. The AfriPop dataset was unsurprisingly near perfect here, as the population data were matched to UNDP population estimates in the modelling procedure. However, our aim was to observe how far away the GRUMP and LandScan datasets were from these most contemporary estimates. Root mean square errors (RMSE) were extracted and differences in population estimates per district were mapped. Secondly, we measured grid-based differences between datasets, as described in Sabesan et al. [23]. Per-pixel absolute differences were mapped and plotted to explore tendencies in these differences. Thirdly, we compared the numbers of people predicted in towns and settlements with known population size. In order to allow the calculation of population predicted in small settlements (smaller than 1 km), the LandScan and GRUMP datasets were resampled to 100 m for this comparison. Pearson correlation coefficients and RMSE between predicted and observed population in towns and settlements were extracted. Finally, we tested the impact of the choice of population dataset on estimates of the population at risk (PAR) of *Plasmodium falciparum *(*Pf*) malaria in Somalia. The AfriPop, LandScan and GRUMP datasets were overlaid on the map of *Pf *malaria endemicity classes for the year 2007 (figure 1) produced by Hay et al. [2] and PAR estimates were extracted.

## Results

### Land cover specific population densities

Per LC population densities derived from Kenya were scaled by 0.21, as this weighting provided the lowest RMSE when predicted population was compared against observed population in towns and settlements from the location dataset with known population. The final population density table used for modelling population distribution in Somalia is presented in table 1.

**Table 1:** Average population densities for each LC class in Somalia

Label	Average population density (people/km^2^)	Data used for population density calculation
Airport	0	/
Bare area	0.36	Africover, Kenya
Cultivated herb	33.97	Africover, Kenya
Cultivated tree	19.54	Africover, Kenya
Industrial area	0	/
Lake	0	/
Natural aquatic	3.60	Africover, Kenya
Natural herb	1.33	Africover, Kenya
Natural shrub	2.03	Africover, Kenya
Natural tree	9.74	Africover, Kenya
Refugee camp	77198.90	UN data on Afgooye corridor, Somalia
River bank	0	/
Rural settlement	2990.05	GTI; settlement points
Sand beach	0	/
Urban area	18302.98	GTI; settlement points

### Population distribution modelling

**Figure 2:**
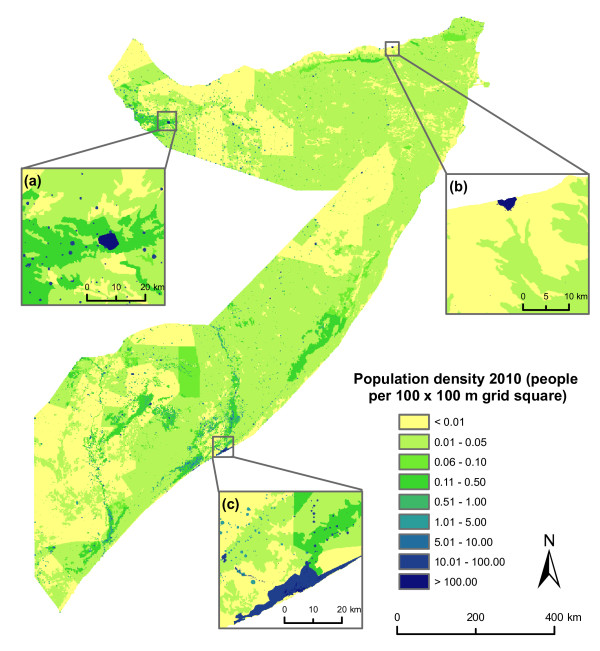
**Population map showing numbers of people residing in each 100 × 100 metre grid square**. Close-ups show detail around (a) Hargeisa, (b) Bosaso, and (c) Mogadishu.

The resulting 2010 gridded population dataset of Somalia at 100 × 100 meter resolution is presented in figure 2. This figure shows that in the vast majority of Somalia, the population density is estimated to be lower than 0.05 people per 100 × 100 meter grid square, i.e. 5 people per square kilometer. Population densities are higher around the two biggest cities - Mogadishu and Hargeisa - and along the Shabelle and Juba rivers. Table 2 shows that 80.7% of the total population is located in towns, settlements and IDP camps in Somalia. The percentage of people living in towns, settlements and IDP camps is lower in the North West zone (63%) because an important part of the area around Hargeisa is agricultural, where population densities are relatively high. The Central and South zone is also partly cultivated, but the large populations of Mogadishu and the IDP camps around the city mean that the effect of the agricultural land on the proportion of people living outside cities and villages is less. The total population in 2010 is estimated to be 8,757,003.

**Table 2:** Population distribution and projected population estimates in Somalia

Zone	% Pop in towns, IDP camps and rural settlements	Total population 2005	Total population 2010
Central & South	82.7	4,810,467	5,619,949
North West	63.1	863,088	1,007,850
North East	83.7	1,828,756	2,129,204

**Total**	**80.7**	**7,502,310**	**8,757,003**

The first column presents the percentage of people living in settlements (towns, IDP camps or rural settlements) for three Somalian administrative zones and the last two columns show population estimates for 2005 and 2010 in these three zones.

### Comparison with existing datasets

**Figure 3:**
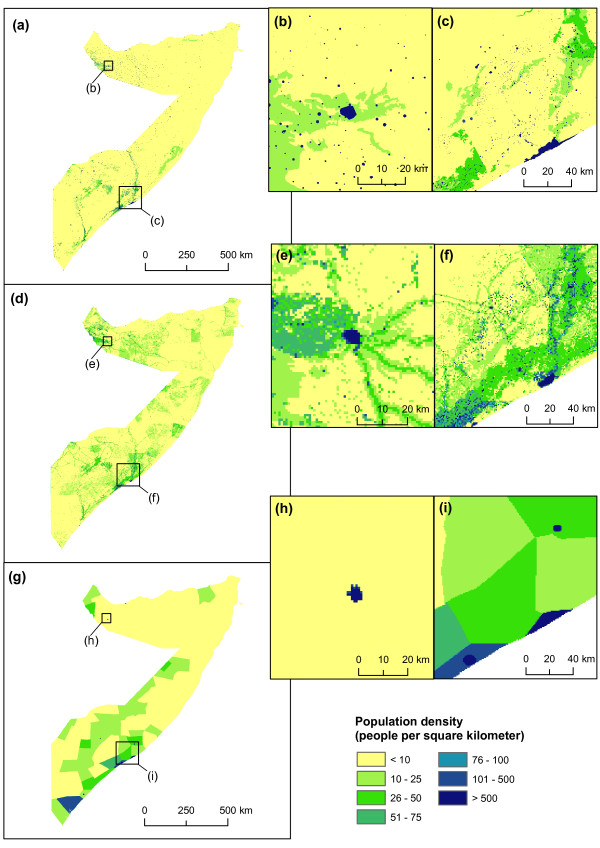
**Comparison of Somalia gridded population datasets**. (a) Newly created population dataset (AfriPop) showing 2009 population numbers in each 100 × 100 metre grid square; (b) close-up of the AfriPop dataset showing detail around Hargeisa; (c) close-up of the AfriPop dataset showing detail around Mogadishu. (d) LandScan 2008 population numbers in each 1 × 1 km grid square [21]; (e) close-up of the LandScan dataset showing detail around Hargeisa (f) Close-up of the LandScan dataset showing detail around Mogadishu. (g) Global Rural Urban Mapping Project (GRUMP) showing 2009 population numbers in each 1 × 1 km grid square [22]; (h) close-up of the GRUMP dataset showing detail around Hargeisa (i) close-up of the GRUMP dataset showing detail around Mogadishu.

**Figure 4:**
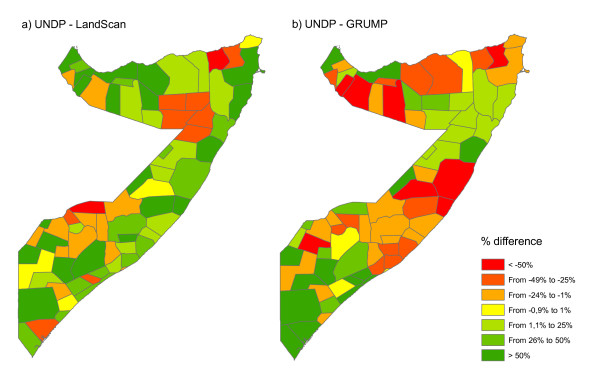
**Differences in population counts per district for the year 2005 (%)**. (a) Difference between UNDP estimates and LandScan estimates, (b) Difference between UNDP estimates and GRUMP estimates.

Figure 3 shows a visual comparison between the AfriPop, LandScan and GRUMP datasets. In the GRUMP dataset, the construction methodology means that population is concentrated into a few major urban areas and areal weighted in the remaining of the districts [24], whereas the new AfriPop dataset shows scattered population clusters in rural areas. In the LandScan dataset, the construction methodology means that populations are clustered around roads and less concentrated in villages and towns, but more diffuse in rural areas. The total population per district predicted by the LandScan dataset is closer to the UNDP estimates than the GRUMP dataset, though overall RMSEs values are relatively high in both cases (40,850 for LandScan and 111,352 for GRUMP). As shown in figure 4, LandScan overestimates population counts compared to the UNDP estimates in most of the districts, whereas GRUMP underestimates these population counts in many districts, principally in eastern central and Northern Somalia. The total population also differs: the UNDP (and consequently the AfriPop dataset) estimated there to be 7.5 million people living in Somalia in 2005, whereas the LandScan and GRUMP population datasets predicted 8.5 and 7.9 million people for the same year, respectively.

**Figure 5:**
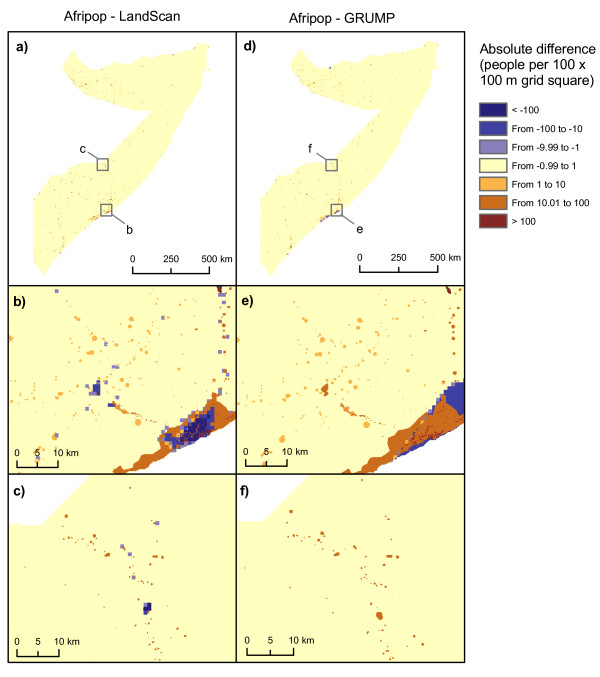
**Per-pixel absolute differences between population datasets**. (a,b,c) Differences between AfriPop and LandScan, and (d,e,f) Differences between AfriPop and GRUMP. Close-ups show detail around the capital Mogadishu (b,e) and the Northern part of the Shabelle river (c,f).

**Figure 6:**
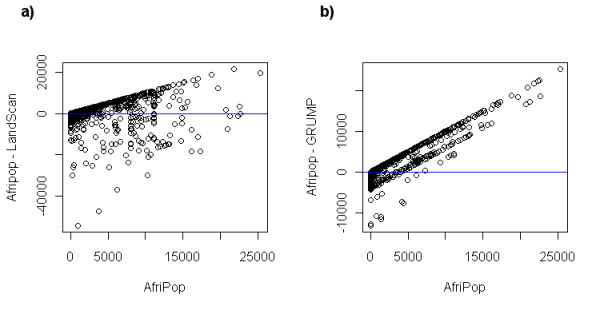
**Absolute differences between population datasets plotted against the AfriPop values**.

Grid-based difference measures led to interesting insights on where the differences between datasets are the most significant. Figure 5 shows that, for the large majority of pixels, the absolute difference between Afripop and LandScan and between AfriPop and GRUMP is lower than 1 person per 100 × 100 m grid square. For these pixels with very low differences, the human population density is generally close to zero. However, the absolute differences can be much higher in more densely populated places. The urban extent of major cities such as Mogadishu strongly influenced the results (figure 5b,e). For the GRUMP dataset, urban extents were derived principally from night-time lights satellite imagery and urban areas are therefore wider in extent, as a result of the 'blooming' effect exhibited by such imagery [11]. As a consequence of the GRUMP methodology, population distributions are more homogenous. The urban extents of the LandScan dataset are generally smaller, resulting in significantly higher population densities in city centers. In more rural regions, such as the Northern part of the Shabelle river, the main difference between the datasets is that more villages and small towns are represented in the AfriPop dataset (figure 5c,f). The LandScan dataset shows some higher density pixels, mainly along only the major roads. Figure 6 shows the relationship between absolute differences and AfriPop values. In this figure, every 1 × 1 km grid square is represented by one point. Points above the line represent grid squares with a positive absolute difference (i.e. a higher population value for AfriPop than LandScan or GRUMP) and points below the line represent grid squares with higher population value for LandScan or GRUMP than AfriPop. Figure 6b shows that the absolute differences between AfriPop and GRUMP are generally positive and increase drastically for large AfriPop values. Figure 6a shows that absolute differences between AfriPop and GRUMP are broader, especially for pixels with a higher value for LandScan than AfriPop. The comparison of population numbers predicted in settlements with independent settlement size estimates showed a better adjustment for AfriPop (Pearson correlation = 0.91; RMSE = 2553), followed by LandScan (Pearson correlation = 0.89; RMSE = 3005) and GRUMP (Pearson correlation = 0.83; RMSE = 4541).

**Figure 7:**
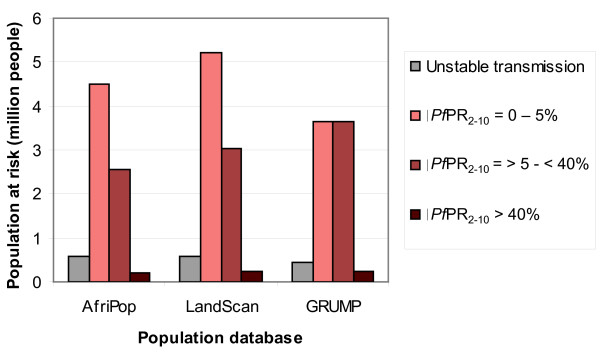
**Population at risk of *Plasmodium falciparum *in Somalia in 2007 according to different population datasets**.

Finally, PAR estimates were extracted from the *Pf *malaria endemicity map shown in figure 1. The PAR was calculated for the 4 endemicity classes defined in [2] and results are presented in figure 7. Important differences can be observed between PAR estimates based on the different population datasets. LandScan predicted higher PAR values than AfriPop, with substantial differences of over half a million in the 0-5% and 5-40% *Pf*PR classes, though the proportion of people in each endemicity class was roughly similar. PAR estimates derived from GRUMP, however, showed significant differences to AfriPop in both PAR sizes and distributions, with over a million more people estimated to be residing in the 5-40% class, and a significantly higher proportion of the population estimated to be residing in the highest transmission areas.

## Discussion

Population movements are particularly intense in Somalia, with currently approximately 1.5 million internally displaced people [1], making the spatial quantification of population distributions a difficult task. Modelling population distribution is however of key importance for estimating the population at risk of infectious diseases, and, ultimately, disease burden. Results presented here show that it is possible to produce contemporary and detailed settlement and population datasets of Somalia using existing detailed datasets and methods that can easily be updated as new data becomes available.

We have used a combination of methods to develop a population distribution model that is matched to administrative population totals provided by the UN. A particular emphasis was given on the integration of settlement extents in the LC dataset. Given that populations in Somalia are highly clustered, and these rich datasets exist, it was important to ensure that the settlements - where the vast majority of Somali people live - were mapped as accurately, and with as much spatial detail, as possible. We used simple and semi-automated methods that can be implemented with free image processing software to produce easily updatable data at 100 × 100 meters spatial resolution. Given the scales and speed with which population movements are occurring in the region, such features are a necessity. The new dataset was compared to existing population datasets. Even if a conclusive and completely fair comparison is not possible due to resolution and construction differences, the results of these analyses allowed the identification of major differences between the datasets. Our tests showed that total population numbers differ and that important differences in population values can be observed in more densely populated places, i.e. in towns and villages. The population density within the main cities strongly depends on the urban extents used in the population mapping procedure. This supports the idea that using accurate and spatially detailed settlement extents is of key importance in population distribution modelling. Thanks to its finer spatial resolution, the AfriPop dataset was able to incorporate data on hundreds of small villages that were not represented in the LandScan and GRUMP datasets.

Major differences observed between the estimated PAR of *Pf *malaria demonstrate how important the choice of population dataset is in disease risk and burden estimations. Our results showed that differences in population distribution can induce large differences in PAR estimates. The AfriPop and LandScan datasets showed a similar partition of people between endemicity classes, but with substantial differences in absolute numbers. The GRUMP dataset, which has been used to estimate PAR of *Pf *malaria in the past [2] predicted a much larger number of people living in highly endemic areas than the more detailed AfriPop dataset.

As discussed previously, determining the accuracy of spatial population datasets is often a difficult operation, given the usage of all available datasets in the interpolation process. Thus, deciding definitively upon which population dataset provides the most accurate estimates of PAR here is impossible. However, it is well known that malaria transmission in Somalia is focal and heterogeneous [25], partially due to the clustered nature of the population distribution. As the precision and detail of malaria transmission mapping improves, spatial population datasets that capture these patterns are therefore required if PAR is to be more accurately quantified. The areal-weighting approach applied to relatively coarse administrative unit level census counts, as was undertaken for production of the GRUMP map (figure 3), fails to capture such patterns. The interpolation approach adopted for the production of LandScan aims to map the clustered nature of population in Somalia, but the completeness of the input data is not clear without the provision of extensive source and metadata information, and previous assessments have suggested that the use of incomplete input data are detrimental to accuracies [5]. By making use of complete, contemporary and well-validated datasets to capture the over dispersed population distribution patterns within Somalia, we therefore have reason to believe that the estimates of PAR derived through the AfriPop dataset constructed here are likely to be the most accurate.

The population distribution modelling approach developed in this paper and others [12,13], will be applied to other low-income sub-Saharan countries. The methodology used will however differ from country to country according to data available; one of the principal aims of the AfriPop project [26] is to make use of detailed, well-validated datasets where they exist to improve mapping precision and accuracy. In most sub-Saharan countries, detailed spatial population data are lacking, but are often of primary importance for disease burden estimation and health service planning. Future work on malaria PAR and burden estimation will rely on these more detailed spatial population datasets, and the potential exists to improve such estimates for other diseases across the continent.

## Conclusions

Detailed and contemporary spatial population data are valuable for assessing the risks and burden of infectious diseases, for planning humanitarian assistance, resource allocation, or public health strategies. The construction of a detailed population database for Somalia has been described here using routinely collected data and semi-automated methods that can easily incorporate new data as it becomes available. The 100 × 100 meters gridded population dataset is freely available as a product of the AfriPop Project [26]. The AfriPop project aims to provide detailed and open access spatial population datasets for all African countries.

## Competing interests

The authors declare that they have no competing interests.

## Authors' contributions

CL and AJT developed the population distribution modelling methodology. CL produced the population map of Somalia, performed the analyses and wrote the first draft of the manuscript. VAA, AMN and RWS participated in data acquisition and interpretation of results. AJT led the design of the study and helped to write the manuscript. All authors read and approved the final manuscript.
